# Ozone Differentially Affects Perception of Plant Volatiles in Western Honey Bees

**DOI:** 10.1007/s10886-016-0717-8

**Published:** 2016-06-25

**Authors:** Stefan Dötterl, Marina Vater, Thomas Rupp, Andreas Held

**Affiliations:** Department of Ecology & Evolution, Plant Ecology, University of Salzburg, 5020 Salzburg, Austria; Department of Plant Systematics, University of Bayreuth, 95440 Bayreuth, Germany; Bayreuth Center of Ecology and Environmental Research, University of Bayreuth, 95440 Bayreuth, Germany

**Keywords:** *Apis mellifera*, Electroantennography, Floral and vegetative volatiles, Atmospheric pollutant

## Abstract

Floral scents play a key role in mediating plant-pollinator interactions. Volatile organic compounds (VOCs) emitted by flowers are used by flower visitors as olfactory cues to locate flowers, both from a distance and at close range. More recently it has been demonstrated that reactive molecules such as ozone can modify or degrade VOCs, and this may impair the communication between plants and their pollinators. However, it is not known whether such reactive molecules also may affect the olfactory system of pollinators, and thus not only influence signal transmission but perception of the signal. In this study, we used electroantennographic measurements to determine the effect of increased levels of ozone on antennal responses in western honey bees (*Apis mellifera* L.). Linalool and 2-phenylethanol, both known to be involved in location of flowers by the bees, and (*Z*)-3-hexenyl acetate, a widespread green leaf volatile also detected by bees, were used. The results showed that ozone affected antennal responses to the different substances differently. Ozone decreased antennal responses to (*Z*)-3-hexenyl acetate, whereas responses to linalool and 2-phenylethanol were not influenced by ozone. Overall, the study does not provide evidence that pollination by honey bees is impaired by damage in the olfactory system of the bees caused by increased levels of ozone, at least when linalool and 2-phenylethanol are the attractive signals. However, the results also suggest that ozone can change the overall perception of an odor blend. This might have negative effects in pollination systems and other organismic interactions mediated by specific ratios of compounds.

## Introduction

Pollination by insects is a key ecosystem service not only in natural but also in managed terrestrial ecosystems (Klein et al. 2007). The economic value of insect pollination is suggested to be worldwide €153 billion per year (Gallai et al. 2009). Floral scents play a crucial role in mediating plant-insect interactions and are used by pollinators to locate flowers (e.g., Dötterl and Vereecken 2010). Pollinators utilize a wide range of scent components both as long distance signals and to discriminate among rewarding and non-rewarding flowers at close range (e.g., Dötterl and Vereecken 2010). Recent studies indicate that chemical communication between plants and their pollinators can be disrupted by pollutants, such as nitrogen oxides (e.g., derived from diesel exhaust) and ozone (Farré-Armengol et al. 2016; Girling et al. 2013; Lusebrink et al. 2015). This is because the oxidative airborne pollutants structurally affect or rapidly degrade floral volatiles (Farré-Armengol et al. 2016; Girling et al. 2013; McFrederick et al. 2009). As a result, the distance over which floral scents can be detected by pollinators is decreased (Farré-Armengol et al. 2016) with the effect that plants might be negatively affected in recruiting insect pollinators, and insects in finding food sources.

Despite the knowledge on the effects of atmospheric pollutants on volatile signals, it is not known whether these compounds can directly affect the olfactory system in insects (McFrederick et al. 2009). The aim of this study was to fill this gap in our knowledge by investigating the effect of ozone fumigation on antennal sensitivity in western honey bees (*Apis mellifera* L.). We used ozone because of its importance as an air pollutant. Furthermore, ozone levels in the troposphere have increased when compared to pre-industrial times, and are assumed to continue to increase (Farré-Armengol et al. 2016, and references therein). The western honey bee was selected as our insect system because it is an important model system for sensory physiology and behavior (Dötterl and Vereecken 2010, and references therein), and the most important pollinator of crops (Klein et al. 2007). Specifically, we asked whether antennal responses to the widespread floral volatiles linalool and 2-phenylethanol, both known to be attractive to honey bees (Dötterl and Vereecken 2010), are influenced by fumigation of antennae with ozone. We additionally tested the common green leaf volatile (*Z*)-3-hexenyl acetate, which elicits defensive behaviors (e.g., stinging) in the bees (Henning et al. 1992).

## Methods and Materials

### Study System

The experiments were carried out using workers of the western honey bee *Apis mellifera* L., collected in the front of bee hives when returning from foraging at the Ecological Botanical Garden, University of Bayreuth.

### Ozone Production and Measurement of Ozone Concentration

Ozone (O_3_) was produced using photolysis of molecular oxygen subjected to UV radiation at a wavelength of 184.9 nm. A mercury Pen-Ray lamp (LSP035, LOT, Leatherhead, UK) provided the required radiation. Ozone concentration of the air passing over the antennae (see below) was continuously measured using a UV photometric Ozone Analyzer (Thermo Scientific (2007), Model 49i Ozone Analyzer, Franklin, MA, USA). Ozone fumigation was performed at a concentration of 1000 ppb. Ozone peak concentrations up to 680 ppb were measured between the 1950s and 1970s in Los Angeles, CA, USA (Bachmann 2007), while today levels are typically less than 100 ppb.

### Electroantennography

The monoterpene linalool (97 %, Sigma-Aldrich) and aliphatic (*Z*)-3-hexenyl acetate (≥ 98 %, Sigma-Aldrich) were used 10 fold diluted (*v*/v), aromatic 2-phenylethanol (≥ 99 %, Sigma-Aldrich) was used 100-fold diluted (*v*/v). Paraffin (Uvasol®, Merck/VWR) was the diluting agent. The concentrations used elicited antennal responses close to the maxima as determined by dose-response measurements (data not shown).

To investigate the effects of ozone on the perception of the three different volatiles by the bees, electrophysiological experiments were carried out by using a standard electroantennographic (EAG) approach. For measurements, the bee antenna (one per individual) was cut at the base and tip, and mounted between glass micropipette electrodes filled with insect ringer (8.0 g/l NaCl, 0.4 g/l KCl, 0.4 g/l CaCl_2_). The electrodes were connected to silver wires. Antennae were stimulated at 2 min intervals. 20 μl of each stimulus were applied on filter paper (Whatman No. 1, 0.4 × 4.0 cm) and then put into a pasteur pipette (15 cm in length). Stimuli were released into a continuous flow of humidified air passing over the antenna with a pulse duration of 0.5 s, and a flow of 10 ml/s regulated by a CS-01 Stimulus Controller (Syntech, Hilversum, Netherlands). Data were recorded by a two-channel universal serial bus acquisition controller (IDAC-2) and analyzed using the software EAGPro 1.0, both provided by Syntech.

### Experimental Design

Each antenna was exposed to three stimulus sequences, whereas the three sequences consisted either of a single compound and paraffin negative controls (e.g., paraffin - 2-phenylethanol - paraffin) or of the three compounds and paraffin controls (e.g., paraffin - linalool - 2-phenylethanol - (*Z*)-3-hexenyl acetate – paraffin). When using all three compounds in a sequence, they were used in randomized order. Paraffin controls were used for the first and last measurements in a sequence. In control antennae, humidified air passed over the antennae (see above) during all three sequences. In treatment antennae, humidified air enriched with ozone was used for the second sequence (antennae were fumigated for 2 min before and during this sequence), whereas sequences 1 and 3 were as in control antennae. Sample sizes (# of antennae used) were as follows: linalool (*N*_treatment_ = 8, *N*_control_ = 10), 2-phenylethanol (*N*_treatment_ = 18, *N*_control_ = 19), (*Z*)-3-hexenyl acetate (*N*_treatment_ = 18, *N*_control_ = 24).

To ensure that the air which continuously passed over the antenna differed only in ozone concentration, the tubing containing the humidified air was split into two arms before unifying them again. Air in one of these arms was radiated by the UV light (see before). The “ozone-free” arm was protected with aluminium foil, which covered the delivery set-up. By switching the light on or off, air enriched or not enriched with ozone was obtained. The flow in the “ozone arm” was regulated with a clamp to adjust the ozone concentration.

### Statistical Analyses

To control for differences in antennal sensitivity among antennae, responses to compounds in the first sequence of each antenna were set to 100 %. Responses to the same compounds in the following sequences are given in percent responses obtained in the first sequence.

Responses in the second and third sequence were analysed by *repeated measures ANOVA* (STATISTICA 7) to test for differences in antennal response between treatment and control antennae. We also considered the sequence effect, and the interaction effect between treatment and sequence. If appropriate, *t-*tests for independent samples were used for *post-hoc* comparisons of responses in treatment and control antennae within a specific sequence. *T-*tests for dependent samples were used for *post-hoc* comparisons to test for a sequence effect within treatment and within control antennae. Data from measurements with one and three stimuli in a sequence were combined for the analyses.

## Results and Discussion

**Fig. 1:**
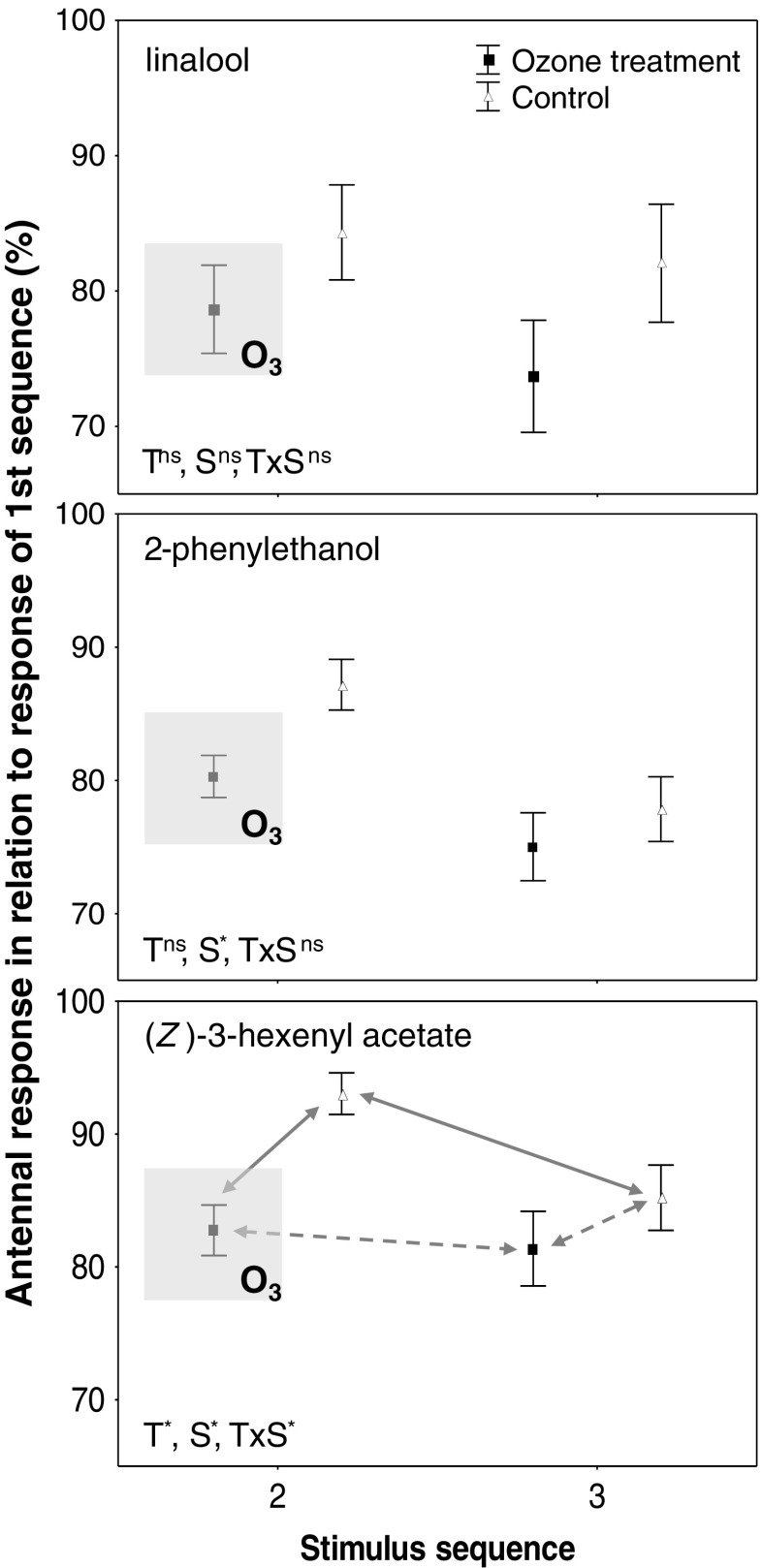
Electroantennographic (EAG) responses (Mean ± SE) of honey bees to the three tested substances, with or without ozone fumigation during the 2nd sequence. None of the antennae were fumigated with ozone during the 3rd sequence. T: statistical treatment effect, S: statistical sequence effect, TxS: statistical interaction effect of T and S; outcomes of a repeated measures ANOVA are given as ns: non-significant (*P* > 0.05) or *: significant at *P* < 0.05. Solid and dashed grey lines show significant (*P* < 0.001) and non-significant (*P* > 0.05) effects, respectively, as indicated by *post-hoc* tests

The electroantennographic experiments revealed that ozone affected antennal responses to different substances differently (Fig. 1). For linalool, no effect of ozone fumigation was detected (*F*_*1*,*16*_ = 2.02, *P* > 0.05), and outcomes were non-significant for the sequence (*F*_*1*,*16*_ = 2.78, *P* > 0.05) and interaction (*F*_*1*,*16*_ = 0.90, *P* > 0.05) effects. Responses to 2-phenylethanol were smaller in the 3rd than the 2nd sequence (*F*_*1*,*35*_ = 31.83, *P* < 0.001), independent of whether antennae were fumigated with ozone or not (treatment: *F*_*1*,*35*_ = 3.04, *P* > 0.05, interaction effect: *F*_*1*,*35*_ = 2.46, *P* > 0.05). Responses to (*Z*)-3-hexenyl acetate were affected by ozone fumigation (*F*_*1*,*40*_ = 5.66, *P* = 0.022), and for this compound we also obtained significant sequence (F_1,40_ = 18.52, *P* < 0.001) and interaction (*F*_*1*,*40*_ = 9.09, *P* = 0.005) effects. *Post-hoc* analyses revealed that antennal responses were reduced only during ozone fumigation, i.e., in the 2nd sequence, whereas responses between control and treatment antennae did not differ in the 3rd sequence. A sequence effect was found only for control antennae, but not for antennae treated with ozone (Fig. 1).

This study shows that ozone fumigation results in a decrease in antennal responses to (*Z*)-3-hexenyl acetate, whereas responses to linalool and 2-phenylethanol were not significantly influenced by ozone, despite a general trend of smaller responses in antennae treated with ozone (Fig. 1). Thus, it seems that traits/structures involved in the detection of different compounds are differently affected by ozone. We only can speculate that ozone oxidizes odorant-binding proteins or olfactory receptors (see also McFrederick et al. 2009), both key proteins in olfaction (Leal 2013), to various extents. Empirical evidence for oxidation by ozone of such proteins is missing thus far, however, studies on other organisms and other tissues have shown that ozone can alter protein structures (e.g., Tognini et al. 1997), making oxidation by ozone of proteins involved in olfaction also likely.

In our experiments, compounds were released into the humidified air, which was or was not enriched with ozone. Thus, ozone also may have oxidized/degraded the volatiles themselves, especially linalool and (*Z*)-3-hexenyl acetate (Atkinson et al. 1995). However, compounds reached the antennae less than a second after having them released in the ozonated air. At 1000 ppb ozone, a fraction of only 1.1 % of the most reactive (towards ozone) compound linalool is degraded within 1 s (Atkinson et al. 1995). Thus, ozonolysis of the compounds was likely not relevant in our study.

We used ozone concentrations rarely reached in nature, and future studies need to show whether detection of volatiles also is influenced by smaller ozone concentrations. Ozone did not affect antennal responses to the two floral volatiles linalool and 2-phenylethanol, despite these high concentrations used. Thus, pollination systems mediated by these compounds would seem not to be impaired by increased levels of ozone causing oxidations in the antennae of honey bees. However, as we found an effect on perception of the aliphatic green-leaf volatile (*Z*)-3-hexenyl acetate, it seems worthwhile to tests for an effect of ozone on perception of other floral volatiles using various concentrations of ozone and various exposure times. Exposure of bees/antennae to continuously increased levels may induce damage not considered in this work (e.g., oxidation of non-antennal proteins). Further, the differential effect of ozone on the sensing of compounds will change the overall perception of an odor blend. This could add another problem to bees and possibly also to other pollinators, especially if relative proportions in a blend play an important role in the processing of the information.

Before our study it was known that volatile-mediated interactions are prone to disruption by air pollutants through direct effects of phytotoxic pollutants on VOC emissions and degradation of VOCs by reactive pollutants in the air (Farré-Armengol et al. 2016, and references therein). We show that direct effects of oxidizing pollutants on the receiving organisms also need to be considered. Relatively minor effects by ozone on pollinators observed in this study might be greater if coupled with a change in volatile emissions through phytotoxicity and a loss of VOCs through degradation.
